# Understanding healthcare workers' experiences of face mask use in healthcare settings during the COVID-19 pandemic: an interview study

**DOI:** 10.1016/j.infpip.2024.100434

**Published:** 2025-01-06

**Authors:** H. Carter, A. Sharp, L. Davidson, C. Foster, E. McGuire, C. Brown, D. Weston

**Affiliations:** a Behavioural Science and Insights Unit, UK Health Security Agency, UK; b Strengthening Project in Zambia, UK Health Security Agency, UK; c Yorkshire and Humber Health Protection Team, UK Health Security Agency, UK; d HCAI, Fungal, AMR, AMU, & Sepsis Division, UK Health Security Agency, UK; e Clinical and Emerging Infections, UK Health Security Agency, UK

**Keywords:** COVID-19, Face masks, Respiratory protective equipment, Healthcare workers, Guidance

## Abstract

**Background:**

Whilst healthcare workers (HCWs) are at high risk of contracting infectious viral diseases, such as COVID-19, measures can be put in place to reduce the spread of diseases in healthcare settings. These currently include the use of different types of masks: fluid-resistant surgical masks (FRSM) and filtering facepiece (FFP3) respirators. However, for mask policies to be effective, compliance with their use must be high.

**Aim:**

To understand any barriers to face mask use, and to promote compliance with face mask policy.

**Methods:**

Twelve HCWs from a variety of backgrounds were interviewed during the COVID-19 pandemic in England in 2022 to understand their experiences of mask use. We explored factors associated with compliance with mask use and potential impacts on HCW wellbeing.

**Findings:**

Overall, participants reported good understanding of the benefits of masks and high compliance levels with policy. However, factors that reduced their compliance with mask policy and impacted their ability to carry out their role were highlighted. These included wearing masks for longer durations, policy being perceived as out of proportion with risk, communication challenges, and discomfort.

**Conclusion:**

This study highlights the importance of clear communication of guidance, particularly when it has changed, ensuring staff are familiar with up-to-date research on efficacy of masks, and ensuring guidance aligns with risk. Furthermore, this study highlights the importance of masks being required for an appropriate duration (based on risk).

## Introduction

Healthcare workers (HCWs) are particularly vulnerable to contracting infectious viral diseases (IVDs), such as severe acute respiratory syndrome coronavirus (SARS), Middle East respiratory syndrome (MERS), and COVID-19 due to close contact with infected patients and contaminated material [1]. Evidence suggests that HCWs are at higher risk of contracting IVDs than the general population [2,3]. COVID-19 is an IVD that caused a recent global pandemic. In April 2020, 6.2% of the National Health Service (NHS) workforce in England was absent and between March 2020 and April 2021 due to sickness, and the proportion of all working days lost due to COVID-19 ranged from 4% to 30% [4,5]. Data demonstrates that HCWs who were more frequently exposed to those with COVID-19, or who work in high-risk settings, have higher infection rates [6].

Measures that can be put in place to reduce the spread of COVID-19 and other IVDs in healthcare settings can be guided by the hierarchy of controls framework and may include physical distancing, ventilation, the use of personal protective equipment (PPE), such as gloves and gowns, fluid resistant surgical masks (FRSM), and filtering facepiece (FFP3) respirators (the term ‘masks’ will refer to both FRSM and FFP3 throughout this paper unless a specific type of mask is being referred to), eye protection, and physical distancing [[7], [8], [9]]. Masks can prevent transmission by reducing the extent to which IVDs are passed on by the wearer (‘source control’) and by protecting the wearer from becoming infected with IVDs (‘wearer protection’) [10]. In the UK, NHS HCWs commonly use both FRSM and FFP3 respirators based on extant national guidance [11]. FRSM usually fit closely on the face and comprise 2–3-ply. They provide one-way protection by capturing micro-organisms leaving the wearer through coughing or sneezing. On the other hand, FFP3 respirators offer a high filtration rate by filtering up to 98% of airborne particles. They provide a tight fit to the face and offer protection to the wearer.

There is broad consensus on using respirators for aerosol-generating procedures (AGPs) [12]. However, outside of AGPs, recommendations for mask use are less specific. International guidance suggests that decisions to use masks should consider factors such as ventilation, the ability of the patient to wear masks, the vaccination status of the patient, the local prevalence of infection, the availability of masks, and HCW preference [[13], [14], [15]]. Masks can also be used either for a single patient interaction (targeted use) or for a period of time when the healthcare worker is undertaking clinical duties in a specific clinical area (sessional use). NHS England guidance advises that mask use should be based on clinical risk assessment, including task being undertaken, patient symptoms, the infectious state of the patient, acquisition risk, and the availability of treatment for the infectious agent [16].

For mask recommendations to be effective, high compliance with recommended policy is essential. During the COVID-19 pandemic, research found that factors reducing compliance with mask use include perceived low risk from COVID-19, perceived low efficacy of masks for reducing the spread of COVID-19, and poor understanding of the rationale for when and why different types of PPE are required [[17], [18], [19], [20]]. Adverse consequences of wearing masks such as discomfort and challenges with communication may also reduce compliance with policy [[21], [22], [23]]. In addition, there is potential for self-contamination if masks are not worn correctly, for example by wearers adjusting the mask with contaminated hands or touching their face or eyes [24]. It is therefore important to understand HCWs' experiences of wearing different types of masks, so that any adverse consequences of mask use can be considered alongside any benefits.

The lack of consensus around face mask use in different healthcare settings, alongside the potential adverse consequences of wearing different types of masks, may reduce compliance with mask use, thereby limiting the efficacy of masks for reducing transmission. It is therefore important to understand any barriers to face mask use, to promote compliance with face mask policy.

This study uses interviews to explore HCW experiences and perceptions of wearing different types of masks, and aims to address the following research questions:

1. What is participants' understanding of current guidance for wearing different types of masks?
2. What are participants' perceptions of the efficacy of different types of masks?
3. What are participants' experiences of wearing different types of masks, in both a targeted and sessional way?
4. What are participants' levels of self-reported compliance with wearing different types of masks?

## Methods

### Design

Semi-structured interviews were carried out to understand participants' experiences and perceptions of wearing different types of masks. Interviews took place over Microsoft Teams between May 27^th^ and August 26^th^, 2022 and lasted between 20 and 62 min.

### Context

The study took place during the height of the COVID-19 pandemic, which was declared a global pandemic on March 11^th^, 2020. At the time of the study, the National Infection Prevention and Control manual had just been published advising on mask use [25]. However, specific protocols for mask use differed between trusts at the time of the study.

### Participants

Participants were HCWs working in one of three different hospital trusts in the UK, referred to as Trust 1 (*N* = 1), 2 (*N* = 7), and 3 (*N* = 4) for anonymity. To identify these trusts, an expression of interest for the study was sent to all English sites that were participating in the SIREN study (https://www.gov.uk/guidance/siren-study). Recruitment materials were sent to a point of contact at each trust that expressed an interest and were subsequently shared with HCWs working in their trust to identify participants. Participants were recruited via convenience sampling and were eligible to take part if they were patient facing HCWs in NHS Trusts in England, working in a clinical area with patients with suspected or confirmed respiratory infections, including emergency departments, acute medical wards, and relevant inpatient wards. Other than two people, everyone who expressed interest in the study and read the information sheet took part. No reason was given why these two people did not take part. Once the interview began, no participants refused to participate, or to answer any questions, and no participants dropped out during the interview. Participant demographics were not collected.

### Materials

A semi-structured interview guide was developed to ensure that key points relating to the research questions were captured whilst allowing participants to share all relevant experiences. The semi-structured interview guide was based on the research questions and contained questions relating to participants': understanding of current guidance for wearing different types of masks; perceptions of the efficacy of different types of masks; levels of compliance with wearing different types of masks; and experiences of wearing different types of masks, for both targeted and sessional use.

### Procedure

Hospitals that had expressed an interest in taking part in the study were contacted and asked to share recruitment information with potential eligible participants. Potential participants were then asked to contact the research team if they were interested in taking part. Those who expressed an interest were asked to sign a consent form and a member of the research team then arranged a convenient time for each participant to take part in an interview. Interviews were carried out by the first, third, and last authors, all behavioural scientists based at the UK Health Security Agency. At the time of the interview all interviewers were qualified to at least MSc level and had received training in qualitative research methods, including carrying out interviews. Interviews were carried out by both male and female members of the research team and only the researcher and the participant were present during the interview. Researchers did not establish a relationship with participants prior to carrying out the interview nor were participants made aware of any personal characteristics of the interviewer, aside from their place of work and the broad aims of the research. Each interview was audio recorded and later transcribed for analysis. After taking part in an interview, participants received a debriefing statement and a £25 voucher to thank them for their participation.

### Analysis

**Figure 1:**
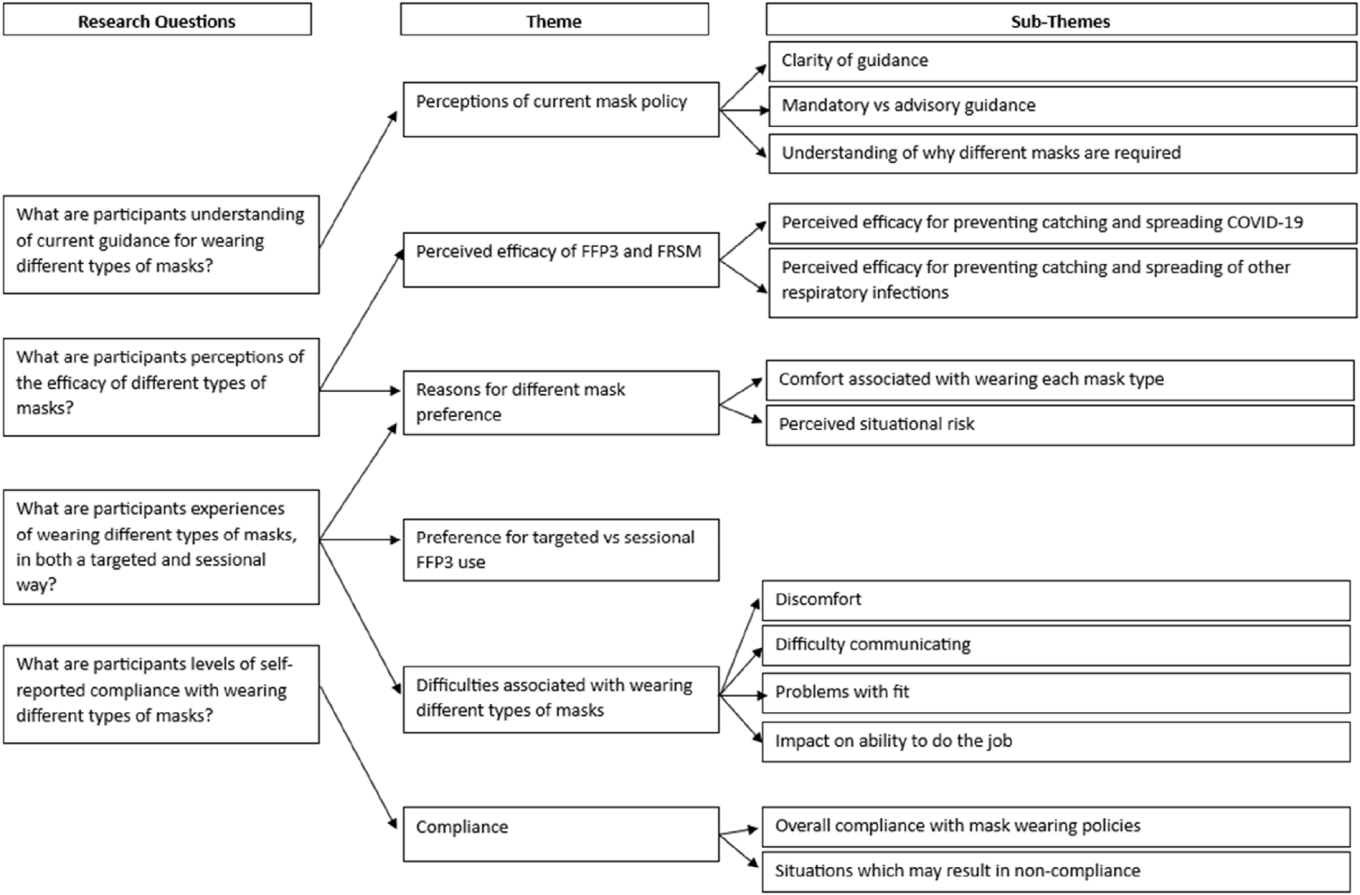
Analytic framework.

Data were analysed using the Framework Approach, a type of thematic analysis which is commonly used in research which has implications for policy and practice [26]. This analytic approach was chosen due to the applied nature of the research with pre-defined research questions which guided the analysis. Analysis followed the five stages of the Framework Approach, starting with familiarisation where the first author read and re-read the transcripts. Next, the first author read the transcripts line-by-line, applying a paraphrase or label (a ‘code’) that described their interpretation of each passage in relation to the research questions. Based on these codes, an initial coding framework was developed. Data were then indexed into broad themes. A coding-by-consensus approach to analysis was used to increase confidence in the dependability and trustworthiness of any results, ensuring any pre-perceptions of the initial coder did not influence the results [27]. This involved the first author sharing initial codes and themes with the research team, which were subsequently discussed and amended until consensus was reached. An analytic framework containing six main themes was then created, agreed by all members of the research team, following which themes were defined and clarified in relation to other themes (see Figure 1). A semi-deductive approach to analysis was used, whereby the six main themes were derived predominantly from the research questions, with the data guiding any sub-themes that were identified in relation to these main themes.

## Results

Twelve participants took part in this study. Six main themes and 13 sub-themes were identified:

1. Perceptions of current mask policy: (i) clarity of guidance; (ii) mandatory vs advisory guidance; and (iii) understanding of why different masks are required.
2. Perceived efficacy of FFP3 and FRSM: (i) perceived efficacy for preventing transmission of COVID-19; and (ii) perceived efficacy for preventing transmission of other respiratory infections.
3. Reasons for different mask preference: (i) comfort associated with wearing each mask type; and (ii) perceived situational risk.
4. Preference for targeted vs sessional FFP3 use.
5. Difficulties associated with wearing different types of masks: (i) discomfort; (ii) difficulty communicating; (iii) problems with fit; and (iv) impact on ability to do the job.
6. Compliance: (i) overall compliance with mask-wearing policies; and (ii) situations which may result in non-compliance.

Quotes are provided and additional quotes can be found in Table I.

**Table I:** Summary of themes, sub-themes, and example participant quotes

Theme	Sub-theme	Example participant quotes
Perceptions of current mask policy	Clarity of guidance	‘The Trust has now changed its guidance three times on what to wear […] no, it's probably not very clear as a Trust’ (P9)
	Mandatory vs advisory guidance	‘It's mandatory […] you know that we have to wear them in these situations, so mandatory’ (P2) ‘To be wearing a mask of some description, I'm sure is mandatory. I just don't know if it's mandatory to wear an FFP3 in certain circumstances or not anymore’ (P4) ‘There might be another setting I go into where it's absolutely mandatory […] it can change ward to ward depending on the type of patient that's been treated’ (P5) ‘I think being told what to wear [is preferable] I think if staff are given an option, then they're always going to go for the one that […] doesn't take as much time up or doesn't take as much energy’ (P7) ‘I'm happy to be told […] but I think that [guidance] needs to be based on sensible recommendations and recommendations that don't try to prevent the spread of COVID everywhere’ (P4) ‘I would like to think I've had enough experience to know which situations to wear which one in’ (P1) ‘I think more long term, perhaps we should be looking at it more in what the practitioner wants to wear themselves’ (P6)
	Understanding of why different masks are required	‘I am aware of the […] rationale as to why we have different types of PPE’ (P6) ‘If we are having any contact with a patient who's COVID positive then we would be required to wear the FFP3’ (P12) ‘Yeah [type of mask is] based on risk exposure, so certain medical procedures and certain devices are more aerosol generating and therefore warrant […] a higher value facemask’ (P10) ‘So we just wear [surgical masks] now around all our corridors, outpatient and clinical areas’ (P7)
Perceived efficacy of FFP3 and surgical masks	Perceived efficacy for preventing catching and spreading COVID-19	‘I think they're effective […] I've got colleagues that have not had COVID for the full two years, and now that we've stopped wearing the masks, we seem to be catching COVID now’ (P3) ‘I'd say it's just as good at keeping the germs in […] a two-way thing’ (P5) ‘I don't think surgical masks have a particularly great impact […] but I do think the FFP3s are definitely worthwhile’ (P6) ‘Surgical masks are a bit more of a transient fit […] they are much tighter fit FFP3s, and I think we know that they're more effective’ (P4) ‘I'd probably say the FFP3 [is more effective] because it's a lot thicker’ (P2)
	Perceived efficacy for preventing catching and spreading of other respiratory infections	‘I think […] they're quite effective across the board’ (P3) ‘I think FFP3s are more effective […] I think surgical masks […] it's not going to be as good as having an FFP3 on’ (P4)
Reasons for different mask preference	Comfort associated with wearing each mask type	‘If I had to have a preference, it would be just a normal surgical mask’ (P3) ‘Surgical masks are nicer, they're easy to wear, they don't feel as full on’ (P1)
	Perceived situational risk	‘Surgical more comfortable, FFP3 more protective, just a risk assessment on wherever I am and what I'm doing’ (P9) ‘If I was going to an area which was higher risk […] I would favour an FFP3 in that situation’ (P6) ‘I'd be quite happy with wearing just the normal surgical masks rather than the FFP3 […] I do think they provide us with enough protection without having to wear an FFP3’ (P3)
Preference for targeted vs sessional FFP3 use	–	‘I do prefer [targeted] I think long term use it's not comfortable’ (P6) ‘I feel a bit safer with [FFP3] on if I'm honest, so I'd prefer to wear those [sessionally]’ (P8) ‘Sessional would probably work better […] I'd say that the effectiveness probably works better having it on for a longer period of time’ (P3) ‘If you use [FFP3] for an extended period of time without changing them […] they won't last indefinitely’ (P1) ‘In the sessional use, I'm forever tempted to just pull it down and have 10 seconds of relief. Whereas if I know in targeted use it's only for 20 minutes, half an hour […] I can cope with it’ (P10) ‘As much as I would probably say that the FFP3's more effective in all settings, and let's all walk around with them all of the time I also don't think that's the best for people’ (P12)
Difficulties associated with wearing different types of masks	Discomfort	‘You can't really breathe with them on, they're really stuffy […] really uncomfortable’ (P2) ‘When you're quite active wearing one all day they can make you quite hot’ (P12) ‘I was wearing them for up to six, seven hours sometimes […] it did start to have quite a bad effect on my skin’ (P1) ‘They do make you feel quite stressed quite […] claustrophobic’ (P3) ‘I would get home at the end of the day and feel really dehydrated with this horrible dry mouth’ (P5) ‘[Surgical masks] are fine […] they're just kind of a normal given every day for me now’ (P10) ‘They're just not nice […] you're thirsty all the time, yeah they're not good’ (P2) ‘The elastic would pull on my ears and I get pain right behind on my skull on both sides’ (P5)
	Difficulty communicating	‘I struggled to hear other people when [they're] wearing an FFP3, or when they're wearing an FFP3’ (P10) ‘You can't communicate properly with [surgical masks] on’ (P8) ‘You weigh it up and you think it's more important that this patient gets an important interaction that he needs than total prevention from COVID’ (P4) ‘[Communication difficulties are] why we got a whiteboard […] but in an emergency that's like you're trying to write something down saying can you go and fetch this' (P2) ‘You end up shouting at the top of your voice at them’ (P8) ‘A lot of patients, I'm having to take down my mask and then say it again’ (P2)
	Problems with fit	‘Sometimes the mask would […] drop down on the face, so that was quite difficult’ (P11) ‘I've got a very small head. That's why I say one size doesn't fit all. So a lot of us had to adjust them’ (P2) ‘[Surgical masks] tend to come down when you're talking […] so you're forever pulling it back up’ (P7)
	Impact on ability to do the job	‘[Wearing an FFP3] would probably make me do what I needed to do perhaps in a quicker manner, so that I was in and out’ (P6) ‘I undertake medical photography, so one of the things we have to do is obviously have the ability to focus through the lens […] trying to do that through a visor with an […] FFP3 mask on is very challenging’ (P5) ‘A lot of my colleagues did struggle because of glasses steaming up’ (P7)
Compliance	Overall compliance with mask wearing policies	‘I've never gone against […] if they say you need to wear one, I will wear one’ (P5) ‘We do follow correct PPE guidelines […] to reduce that risk of infection’ (P7) ‘I chose to actually wear a mask even though I didn't have to […] because I was in very close proximity of people who were coughing’ (P5)
	Situations which may result in non-compliance	‘We've become a little bit more, can I use the word slack […] in an area where there's no patients' (P4) ‘I've got no one sat next to me this afternoon so I've taken my mask off’ (P3) ‘When we're having a conversation on the phone, or we feel like someone can't hear us we pull them down’ (P4) ‘It's sometimes difficult in the moment to don/doff properly when it's an emergency’ (P10) ‘I was probably cutting corners and pulling it off my face because I just couldn't cope with it being strapped so tight’ (P8)

### Perceptions of current mask policy

Participants were asked how they felt about current policy for wearing different masks within their hospital trusts, including whether the guidance is clear and whether it is mandatory or advisory. Within this theme, three sub-themes were identified: clarity of guidance; perceptions of mandatory vs advisory guidance; and understanding of why different masks are required.

#### Clarity of guidance

Participants expressed different views about whether guidance on mask wearing was clear within their hospitals. Some participants felt that the guidance for mask wearing was clear, e.g. ‘it breaks it down very, very clearly into if you're doing this procedure, you need this mask’ (P10)’. However, some participants suggested that the guidance could be clearer. This was particularly the case when guidance changed rapidly or when moving to different areas of the hospital in which different policies were in place, e.g. ‘it's not clear […] I don't know if it could be made clear because it changes all the time, and all the wards change’ (P8).

#### Mandatory vs advisory guidance

Views on whether the guidance was mandatory or advisory were not consistent among participants. Whilst most participants stated that guidance was currently mandatory, some said that guidance in their Trust was now voluntary, e.g. ‘the guidance at the moment is that if you want to have a mask on that's down to you’ (P5). However, a few were unsure whether guidance was mandatory or advisory or said that this can change depending on where they are working in the hospital, e.g. ‘There might be another setting I go into where it's absolutely mandatory […] it can change ward to ward depending on the type of patient that's been treated’ (P5).

Participants differed in their views on whether they would prefer guidance to be mandatory or advisory. The main reason participants gave for preferring guidance to be mandatory was that they did not trust others to choose the appropriate type of mask. Some suggested that mandatory guidance was preferable, but only if it was based on sensible recommendations. Interestingly, while some felt that mandatory guidance would encourage people to adhere to mask policies, it was also suggested that people may be more likely to follow the guidance if it were advisory, e.g. ‘I'd prefer it to be a kind of guided but make your own decision […] I think people are more likely to follow this guidance […] if that's the option as opposed to be compulsory’ (P10). Other reasons given for preferring advisory guidance were that people felt confident in their ability to choose the correct mask, and that mandatory guidance is not appropriate in the longer term.

#### Understanding of why different masks are required

All participants stated that they understood why different masks are required in different circumstances, despite some inaccuracies in understanding of the underlying science, e.g. ‘the surgical masks I understand a bit more general […] but the FFP3's obviously a bit of a better filter to stop the spores coming through from the infection’ (P12). Participants stated that FFP3 masks are required in situations of higher risk, for example when working on a ward with COVID-19 or other respiratory infections, or when performing high-risk procedures. In contrast, participants understood that surgical masks are required in situations with lower risk of transmission, such as general clinical areas (as opposed to those with a high risk of COVID-19).

#### Perceived efficacy of FFP3 and surgical masks

When participants were asked about the perceived efficacy of surgical and FFP3 masks for preventing infection transmission, there were two sub-themes: efficacy for wearer protection and source control of COVID-19; and efficacy for wearer protection and source control of other respiratory infections.

#### Perceived efficacy for preventing catching and spreading COVID-19

Most participants felt that both surgical and FFP3 masks were effective for preventing them from becoming infected with COVID-19. However, some participants felt that both surgical and FFP3 masks had limited efficacy for preventing them from becoming infected with COVID-19, e.g. ‘myself and my colleagues […] we've constantly worn masks and we've still all managed to get COVID’ (P7).

Most participants also perceived both surgical and FFP3 masks to be effective, to some extent, for reducing the risk of them transmitting COVID-19 to others. However, some participants highlighted that reduced transmission of infection also depended on other types of protective actions, rather than solely mask wearing, e.g. ‘wearing the mask will stop you, you know, from spreading the droplets but if you've already coughed in your hand previously and not washed your hand and then touched somebody's face […] that's not going to reduce the risk is it?’ (P7).

The majority of participants perceived FFP3 masks to be more effective than surgical masks for wearer protection and source control of COVID-19. Reasons given for greater perceived efficacy of FFP3 masks included FFP3 masks having a better fit and being thicker.

### Perceived efficacy for preventing catching and spreading of other respiratory infections

As with perceived efficacy for wearer protection and source control of COVID-19, most participants perceived the masks to be effective for wearer protection and source control of other respiratory infections, e.g. ‘if [the mask is] a barrier to stop COVID it must be doing its job, it must be pretty, pretty good at stopping everything else’ (P5). However, some were unsure as to the effectiveness of masks for preventing transmission of other respiratory infections, e.g. ‘I wouldn't know [how effective they are] because I have been at work with colds and things and other people have got colds' (P2). As with COVID-19, most participants perceived FFP3 masks to be more effective than surgical masks for wearer protection and source control of other types of infections.

### Reasons for different mask preference

There were two main factors that affected participants' preference for wearing surgical or FFP3 masks: how comfortable each mask type is to wear; and perceived situational risk.

#### Comfort associated with wearing each mask type

Most participants stated that they preferred to wear surgical masks than FFP3 masks. The main reasons for this were that surgical masks are more comfortable and better for wellbeing than FFP3 masks, e.g. ‘there is the kind of wellbeing and occupational aspect for staff, which has been quite impacting to have to wear an FFP3 for every single patient’ (P4).

#### Perceived situational risk

While most participants generally preferred to wear surgical masks, their preference for different mask types varied depending on the perceived risk of the situation. Several participants stated that, though they prefer wearing surgical masks, they would prefer to wear FFP3 masks in high-risk situations. This was not the case for all, however, with other participants feeling that surgical masks provided sufficient protection or that the risk from COVID-19 has now decreased sufficiently that FFP3 masks should only be used in very specific circumstances, e.g. ‘why would we now be trying to prevent COVID on the scale that we prevented it during the last two years […] I'm very happy to wear FFP3 masks […] where there's a real need and a patient benefit for it, but […] I'm happy enough to wear the surgical mask’ (P4).

### Preference for targeted vs sessional FFP3 use

When asked whether they would rather wear FFP3 masks in a targeted or a sessional manner, the majority of participants stated that they prefer targeted to sessional use. The main reason given for this was the discomfort associated with wearing FFP3 masks for long periods of time. It was also suggested that the risk posed by COVID-19 is now not sufficient to require sessional FFP3 use, e.g. ‘the only way I would wear an FFP3 mask again all day long is if we get a new infectious disease, never again for COVID’ (P4). However, not all participants preferred to wear FFP3 masks in a targeted way, with the main reason given for preferring sessional use being that they felt safer wearing FFP3 sessionally. This participant also noted that it could be frustrating to continually have to change the type of mask they were wearing, e.g. ‘I just think can't be bothered with the faff, just let me wear this, the FFP3, and I'll just keep it on until I need food or drink’ (P8).

Interestingly, although the majority of participants preferred to wear FFP3 in a targeted way, several participants felt that sessional use would be more effective, because it involves wearing the mask for a longer period of time, and continually changing the mask risks contamination, e.g. ‘Unless you're meticulous taking your mask off, you're probably infecting yourself just keep touching your face all the time’ (P8). In contrast, some participants felt that targeted use would be more effective. One reason for this was that participants perceived FFP3 to become less effective if worn for long periods of time. Another reason that targeted use was perceived to be more effective was that participants felt that people would be more likely to touch FFP3 masks (due to discomfort), creating a risk of infection, e.g. ‘In the sessional use, I'm forever tempted to just pull it down and have 10 seconds of relief. Whereas if I know in targeted use it's only for 20 minutes, half an hour […] I can cope with it’ (P10). One participant specifically highlighted that while sessional use might be more effective, wearing an FFP3 mask for long periods of time would not be in people's best interest.

### Difficulties associated with wearing different types of masks

When asked about any difficulties associated with wearing surgical and FFP3 masks, participants identified several issues, which fell into four broad categories: discomfort associated with wearing masks; difficulty communicating when wearing a mask; problems with mask fit; and impact of masks on participants' ability to do their job.

#### Discomfort

Participants described several ways in which they experience discomfort associated with wearing FFP3 masks. These included: difficulty breathing; problems with overheating; problems with skin; feeling claustrophobic; and becoming dehydrated.

Participants reported less discomfort associated with surgical masks. However, some did report some issues including increased dehydration, and headaches caused by tight elastic.

#### Difficulty communicating

Participants highlighted that there were communication difficulties associated with wearing both FFP3 masks and surgical masks. It was suggested that communication is particularly difficult with those who are vulnerable or otherwise have difficulty communicating, e.g. ‘you're trying to use your eyes, you're trying to communicate with your hands […] it was really difficult sometimes, especially some vulnerable or elderly patients who are harder hearing’ (P3), and that at times, the need to interact properly with a patient outweighed the risk of infection. Participants also spoke about ways in which they tried to overcome difficulties with communication, such as writing things down, raising their voice, or partially removing the mask.

#### Problems with fit

Some participants highlighted difficulties associated with mask fit, particularly in relation to FFP3 masks. Difficulties included that masks would sometimes fall down, that FFP3 masks fit some people better than others, and that masks that participants had been fit-tested for sometimes ran out, requiring them to then use a mask they had not been fit-tested for, e.g. ‘sometimes the masks that we were fit-tested on would run out and we'd just use […] one that we were not fit-tested on which would put us at risk’ (P11). Issues associated with fit of surgical masks were reported less commonly (likely because there is less emphasis on the importance of good fit in relation to surgical masks).

#### Impact on ability to do the job

As noted above, participants highlighted that discomfort and communication challenges sometimes impacted their ability to do their job. Participants also highlighted other ways in which wearing an FFP3 mask made doing their job more difficult, including impact on patient care; and difficulty with physically being able to carry out tasks. While the impact of surgical masks was perceived to be less, some participants highlighted issues that were common to both types of masks, such as glasses fogging up and affecting their ability to see.

#### Compliance

When asked about their compliance with mask-wearing policies, participants reported two separate aspects associated with compliance: their overall compliance with mask-wearing policies; and situations which may result in themselves or others being less compliant.

#### Overall compliance with mask-wearing policies

The majority of participants said they were compliant with mask-wearing policies, both for FFP3 masks and surgical masks. Reasons for complying included that mask policies are in place for a reason and that it is safer to follow the guidance, e.g. ‘I would always wear one […] because I know that on our ward, we're only recommending them when they're really needed’ (P4). Indeed, one participant noted that they sometimes chose to wear a mask even in situations in which they were not required, if they felt it was appropriate based on the level of risk.

However, there was some suggestion that compliance generally had decreased over the course of the pandemic, the main reason being that the risk had changed, e.g. ‘Because people are vaccinated, we are relaxed […] we are more relaxed in wearing FFP3 masks than before’ (P11).

#### Situations which may result in non-compliance

Whilst most participants stated that they were compliant with mask-wearing policies, they did identify some circumstances under which they would be less likely to comply. A key circumstance under which participants felt compliance would be lower is when the situation was perceived to be lower risk. Factors which contributed to a perception of a situation as low risk included there being no patients around and people being socially distanced. Other circumstances under which participants felt compliance may be lower included situations in which communication is challenging, and emergency situations in which there may not be time to don or doff the appropriate mask, e.g. ‘When we're having a conversation on the phone, or we feel like someone can't hear us we pull them down’ (P4). Some also stated that discomfort was a reason for non-compliance.

## Discussion

This study explored HCWs' experiences and perceptions of wearing different types of masks in healthcare settings, focusing on the use of FFP3 and surgical masks. It examined participants' understanding of mask guidance, their views on the effectiveness of different mask types, their experiences with wearing masks both in a targeted and sessional manner, and their self-reported compliance with mask wearing.

Findings show that participants generally understood when and why different masks were required based on existing guidance and they recognized that higher-risk situations warranted use of FFP3 masks. This understanding is consistent with both UK and international guidance, which recommends wearing FFP3 respirators during high-risk procedures and in high-risk environments [[13], [14], [15]]. However, participants also found the guidelines challenging to follow due to frequent changes in guidance, a factor that has been linked to reduced compliance in other studies associating poor understanding of mask policies with lower adherence [17]. Therefore, clear communication of updated guidance on mask policy is essential, especially when adjustments are made in response to policy changes. However, this study did not specifically explore where participants obtained their information about mask guidance from, and so future research would benefit from exploring this.

There was no clear consensus among participants on whether mask guidance should be advisory or mandatory. Interestingly, whilst most participants reported that they themselves complied with mask guidance, they believed others were less likely to comply, leading some to prefer mandatory mask-wearing policies. However, participants were more aligned with their preference of using FFP3 masks in a targeted rather than sessional way. This was due to the discomfort associated with wearing these masks for an extended duration that impacted both participants' ability to do their job and their wellbeing. This is in line with previous research demonstrating that masks have the potential to result in discomfort [22,28,29]. Interestingly, participants suggested that the discomfort associated with prolonged FFP3 use may result in them touching or interfering with their FFP3 mask, potentially reducing its effectiveness. However, a systematic review of 19 randomized control trials found that respirators were effective at preventing the transmission of COVID-19 if worn continually during a shift but were not effective at preventing transmission if worn intermittently [30]. Therefore, future studies would benefit from exploring the potential impact of interference with FFP3 respirators on transmission, exploring the relative efficacy of good adherence to targeted FFP3 use compared to poor adherence to sessional use.

Most participants said they would follow mask guidance because they perceived masks to be effective in preventing COVID-19 and other respiratory viruses. In addition, participants believed FFP3 masks were more effective than surgical masks in preventing COVID-19 and other respiratory viruses. While this is in line with pblished research, participants often based these views on their personal experiences [31]. Many participants had not caught COVID-19 whilst wearing FFP3 masks, reinforcing this belief that FFP3 masks are more effective than surgical masks. However, this reliance on personal experiences also introduced some uncertainty about the overall effectiveness of masks, particularly if they or someone they knew had contracted COVID-19. Since perceived efficacy promotes compliance, it is important that HCWs view masks as effective [19]. Providing them with up-to-date research on the efficacy of different mask types could help improve compliance with guidance. Our study also highlighted that compliance may be improved when guidance is perceived to be proportionate to risk. These findings highlight the importance of communicating not only what the guidance is but also the level of risk. This is in line with previous research showing that misinformation around the origin, severity, and prevention of COVID-19 was a key factor for non-compliance with preventative behaviours [32].

In line with previous research, this study highlighted that there were situations where participants actively chose not to comply with guidance. Reasons for this reduced compliance included difficulties communicating with patients when wearing masks, particularly with patients who were vulnerable or who faced additional communication challenges, and discomfort associated with wearing masks (in particular FFP3 masks). This is in line with previous research suggesting that masks can hinder communication both in those with and without a hearing impairment and that discomfort associated with mask wear is a key factor in reducing compliance with guidance [19,22,33]. We also found that practical issues, such as masks frequently needing adjustment and leading to unavoidable face-touching, sometimes made it physically difficult to comply with guidance. Interestingly, previous research suggests that risk aversion and social preferences (e.g. wanting to protect others) may also play a key role in motivating HCWs' compliance with mask wearing [34]. While we did not find evidence of this in the current study, it may be beneficial for future research to explore such factors further.

This study provides an in-depth exploration of mask use among HCWs across England, offering important insights for policy and practice. However, despite the strengths of the study, there are limitations that need to be addressed. First, participants opted into this study, and it is therefore possible that the views of those who opted into the study differ from the views of those who did not. This therefore may potentially bias the findings towards the experiences and views of the current sample, rather than the general HCW population. A further potential bias of the sample is that while participants worked in various hospital settings, they were recruited from only three hospitals. Furthermore, the sample size of 12 is a potential limiting factor of this study. This study took place while the COVID-19 pandemic was ongoing, a time when healthcare systems and front-line workers were under immense pressure. Given these circumstances, we aimed to recruit as many participants as possible within the constraints of the situation. Despite this, a sample size of 12 for an in-depth interview study is in line with recommendations for qualitative research, especially for hard-to-reach specialist participants, as in the current study [35,36]. It is therefore important that our findings are considered alongside other research examining HCWs' experiences and perceptions of masks, and that broader research across more hospitals is conducted.

While interviews were conducted in late spring/summer of 2022 (a relatively stable COVID-19 period in the UK) it is possible that guidance changes across the three months of data collection may have impacted HCWs' perceptions of masks. Findings may therefore lack generalizability to other HCW contexts or periods of time within which mask use is less variable. Additionally, the relevance of the current study to contexts not involving HCWs may be limited due to HCWs being less constrained by practical barriers, such as access to masks, than the general population [34]. Therefore, in order to extrapolate findings to wider non-HCW contexts, future research on perceptions of mask use in the general population is required.

Whilst measures were taken to reduce researcher bias in this study through the use of coding-by-consensus approach to analysis, there is a possibility that bias is still present in the findings [27]. Therefore, findings should be considered alongside other relevant research. Finally, this study relied on self-report descriptions of mask use and adherence to guidance which might introduce bias. As such, future research would benefit from developing an observational tool for mask use.

## Conclusion and recommendations

Overall, the HCWs in this study reported good understanding of the benefits of FFP3 masks and high levels of compliance with mask policy. However, they highlighted some factors that reduced their compliance with mask policy and impacted their ability to carry out their role. These included wearing masks for longer durations (sessional as opposed to targeted use), guidance being perceived as out of step with risk, and communication challenges and discomfort associated with mask use. Based on these findings, to improve compliance to future mask policy, we recommend:

1. Mask guidance should be communicated to all staff clearly, particularly when it has changed, for example through internal, quality-approved systems.
2. Guidance should include references to relevant and up-to-date research on the efficacy of masks to promote greater staff understanding and avoid reliance on personal experiences.
3. Mask guidance should be aligned with risk levels and this risk level should be communicated to staff.
4. Mask guidance should include details on how long masks should be worn for, and this duration should be proportional to risk.
5. Policymakers should consider user and patient experience, concerns, and lived experience of end users when developing guidance.

## Data availability

Data are available upon reasonable request.

## CRediT author statement

**Holly Carter**: Conceptualization, Methodology, Formal analysis, Investigation, Writing - Original Draft, Writing - Review & Editing. **Ashley Sharp**: Conceptualization, Methodology, Formal analysis, Investigation, Writing - Review & Editing. **Louise Davidson**: Conceptualization, Methodology, Formal analysis, Investigation, Writing - Review & Editing. **Clare Foster**: Conceptualization, Methodology, Formal analysis, Investigation, Writing - Review & Editing. **Emma McGuire**: Conceptualization, Methodology, Formal analysis, Investigation, Writing - Review & Editing. **Colin Brown**: Conceptualization, Methodology, Formal analysis, Investigation, Writing - Review & Editing. **Dale Weston**: Conceptualization, Methodology, Formal analysis, Investigation, Writing - Review & Editing.

## Funding sources

No specific funding was received for this research. All authors who carried out this research were funded by the UK Government, with some authors funded via Health Protection Research Units (as noted within acknowledgements) and others funded directly from the UK Health Security Agency. No private funding was received for this research.

## Conflict of interest statement

None declared.
